# Impact of Occlusal Overloads on Complications in Fixed Prosthetic Dentures

**DOI:** 10.3390/jcm14186388

**Published:** 2025-09-10

**Authors:** Diana Elena Vlăduțu, Angelica Diana Popa, Alin Gabriel Ionescu, Maria Filoftea Mercuț, Mihaela Roxana Brătoiu, Ileana Cristiana Petcu, Maria Alexandra Rădoi, Adrian Marcel Popescu, Veronica Mercuț, Monica Scrieciu, Mihaela Ionescu, Alexandru Ștefîrță

**Affiliations:** 1 Department of Prosthetic Dentistry, University of Medicine and Pharmacy of Craiova, 200349 Craiova, Romania; diana.vladutu@umfcv.ro (D.E.V.); cristiana.petcu@umfcv.ro (I.C.P.); alexandra.radoi@umfcv.ro (M.A.R.); smpopescu@mail.com (A.M.P.); veronica.mercut@umfcv.ro (V.M.); monica.scrieciu@umfcv.ro (M.S.); alexandru.stefirta@umfcv.ro (A.Ș.); 2 Doctoral School, University of Medicine and Pharmacy of Craiova, 200349 Craiova, Romania; dr.diana_popa84@yahoo.com; 3 Department of History of Medicine, University of Medicine and Pharmacy of Craiova, 200349 Craiova, Romania; 4 Department of Ophthalmology, University of Medicine and Pharmacy of Craiova, 200349 Craiova, Romania; 5 Department of Medical Informatics and Biostatistics, University of Medicine and Pharmacy of Craiova, 200349 Craiova, Romania; mihaela.ionescu@umfcv.ro

**Keywords:** fixed prosthetic dentures, occlusal overloads, complications, metal–ceramic, metal–acrylic, abutment teeth, cantilever pontics

## Abstract

**Background/Objectives**: Fixed prosthetic dentures (FPD) represent a reliable treatment option to rehabilitate oral function and aesthetics. The concentration of loads at a certain level of the bridge causes overloading of the bridge components. The objectives of this study were to identify the frequency and severity of complications in FPDs associated with occlusal overload for patients prosthetically treated with metal–ceramic and metal–acrylic FPDs. **Methods**: This retrospective cross-sectional clinical study included 306 patients of both sexes, aged 30+, with an FPD requiring rehabilitation. The following data were collected: general demographic data of the patients, data on dental bridges, data on occlusal overloads, and complications of FPDs according to severity. Statistical processing was performed in SPSS. **Results**: Metal–ceramic FPDs had more abutment teeth (*p* = 0.035), fewer intermediaries (*p* < 0.0005), and less severe complications (*p* = 0.001). Maxillary FPDs had a higher number of abutment teeth, compared to mandible FPDs (*p* < 0.001). As the life duration of the FPDs increased, the number of intermediaries and the severity grade also increased (*p* < 0.001). Compared to intercalated and pontics, cantilever pontics of FPDs had a longer duration (*p* < 0.001), a lower number of intermediaries (*p* < 0.001), and a higher severity grade (*p* < 0.001). The presence of occlusal interferences was associated only with a lower FPD duration (*p* = 0.007). The presence of unbalanced occlusion planes was associated with a higher severity grade, while the presence of shortened arches was associated with a longer FPD duration (*p* < 0.001), a higher number of intermediaries (*p* = 0.005), and a higher severity grade (*p* < 0.001). **Conclusions**: The most severe complications of FPDs were recorded in the case of shortened arches and cantilever bridges. This study draws attention to the need for preprosthetic and postprosthetic occlusal balancing and the need for complete rehabilitation of the dental arches.

## 1. Introduction

Oral diseases, especially the impairment of the dento-maxillary system (DMS) functions, play a crucial role in systemic health [1]. Oral diseases are generally associated not only with the quality of life related to oral health but also with the general quality of life, defined as “an individual’s perception of his or her position in life in the context of the culture and value systems in which he or she lives and in relation to his or her goals, expectations, standards and concerns” [2].

Oral rehabilitation is the restoration of the morphology, function, and aesthetics of the masticatory system [3]. The main objectives of oro-dental treatments are represented by the restoration of the morpho-functional balance of the DMS and the education of the patient in order to maintain this balance associated with optimal oral health [4].

Oral rehabilitation intended to improve DMS functions includes operative dentistry treatments, periodontal therapy, and prosthetic treatments [5]. Rosenstiel 2023 stated that the prosthetic treatment planning process addresses four objectives: the patient’s primary desire, treatment of any existing dental disease, improvement of function, occlusion and aesthetics, and prevention of future disease by instilling a lifelong attitude of professional and personal maintenance [6]. Due to the complexity of prosthetic treatments, the patient must be aware of the advantages, disadvantages, alternatives, limitations, costs, and complications of prosthetic treatments.

In the case of fixed prosthetic dentures (FPDs) treatments, the long-term outcome depends not only on closing the edentulous gap and resolving its complications but also requires control of the biomechanical behavior of the prosthetic restoration and of the support of the dental bridge represented by the abutment teeth [7].

The FPD treatment plan must be designed so that the restoration restores the morpho-functionality of the DMS and withstands the stresses caused by occlusal forces and span length, so that premature failure through fracture of the physiognomic veneering material, fracture of the bridge framework, loss of retention, tooth mobility, or an unfavorable tissue response are avoided [6]. A series of stresses develop within dental bridges in the form of tilting, torsion, and bending movements caused by the mechanical properties of the dental materials, the cross-sectional shape, and length of the bridge, which are transmitted to both the restoration and the abutment teeth [7].

Occlusal loads characterized by amplitude, duration, frequency, and direction, distributed along the arch, influence the evolution of dental restorations and abutment teeth [7].

A number of particular and pathological situations can determine occlusal loads to cause overstressing of prosthetic restorations. Gordon Christensen, in 2005, showed that the assessment of occlusion and the treatment of pathological or potentially pathogenic occlusal conditions are the most neglected areas of dentistry [8]. He listed several situations in which he considered occlusal balancing to be necessary as follows: in the case of premature contacts, after orthodontic treatment, in the treatment of bruxism, for aesthetic reasons (unbalanced occlusal plane), in the treatment of temporomandibular disorders [TMD], and in periodontal therapy [8]. In 2017, Nicolas Patricio Skármeta stated that, although some time has passed since Christensen’s observations regarding the importance of occlusal balancing, a large portion of doctors still neglect it [9].

On the other hand, most prosthetic and odontological restorative procedures affect the shape of the occlusal surfaces [6]. Any dental treatment, but especially the rehabilitation of the occlusal surfaces of the dental arches, no matter how correct it is, modifies the occlusal morphology, the distribution of occlusal forces, and even the static and dynamic occlusal relationships [10]. Prosthetic restorations that fit the occlusal morphology and functionality show a better evolution compared to those in over-occlusion or those that show interference during mandibular movements, which exert non-physiological loading prosthetic restorations [11]. When occlusal forces are higher, common failures of the restoration include fractures, chipping, decimation, or wear, especially in the posterior regions [12]. The clinical effects on restorations in the case of improper occlusion can lead to localized stress concentration, leading to microcracks or decimation over time [13].

The objectives of this study were to determine the frequency and severity of FPD complications associated with occlusal overload in a group of patients prosthetically treated with metal–ceramic and metal–acrylic FPDs, testing the hypotheses that the occlusion is not an important factor in the evolution of the dental bridges.

## 2. Materials and Methods

### 2.1. Study Design

This retrospective cross-sectional study was conducted between 10 January 2023 and 30 December 2024 on a group of 428 patients who presented to the Dental Prosthetics Clinic, within the University of Medicine and Pharmacy of Craiova (UMFCV), for various complications of dental bridges. Since the present study is retrospective, the confirmation of a minimum sample size would ensure that the reported findings are reliable. This value was computed using the software application G*Power 3.1.9.7 (Heinrich Heine University Düsseldorf, Germany) for multiple test families, based on the following parameters: a power 1 − β equal to 0.95, a significance level α of 0.05, and a medium effect size value of 0.3 (focusing on an awareness of practical significance), thus resulting in a minimum of 243 patients.

This study was approved by the University Ethics and Deontology Committee of UMFCV, No. 234 of 7 December 2022, and was conducted in compliance with the Declaration of Helsinki [14]. Previously, written informed consent was obtained from all study participants.

### 2.2. Patients’ Selection

The inclusion criteria in the study were patients of both genders, with ages more than 30 years old, who had at least one conventional FPD with complications, and who gave their consent to be included in the study (after the acknowledgment of the informed consent).

The exclusion criteria in the study were patients who did not give their consent for inclusion in the study, patients with incomplete data in the patient’s charts, patients without FPDs or with another type of prosthetic restoration than FPD, and patients with communication and neuromuscular coordination deficiencies.

### 2.3. Patient Examination

For patients included in this study, the treatment records in the clinic database were consulted; for new patients, a treatment record was prepared based on a thorough anamnesis. Subsequently, the patients were clinically examined and an orthopantomography was performed.

The intraoral examination was performed using the standard technique of inspection, palpation, percussion, and probing, respecting the standards of the World Health Organization and the Declaration of Helsinki [14,15], together with the imaging evaluation based on orthopantomography by the same dental specialist. The dental bridges were examined at the level of the aggregation elements and the bridge body to establish the form of the complication and its severity. The dental occlusion was evaluated statically and dynamically to highlight premature contacts and occlusal interferences. Their identification was performed with the help of articulation paper. The unevenness of the occlusal plane was highlighted by inspection of the curves, the curve of Spee, the curve of Wilson, and the incisal curve. The arches were considered shortened when less than 20 teeth were present in the oral cavity [16], possibly in the absence of molars.

### 2.4. Data Collection and Definition of Variables

General data about patients were recorded: age, gender, and residence. The following age groups were defined: 30–45 years corresponding to adult age; 46–60 years corresponding to transitional age; 61–75 years corresponding to elderly; and over 75 years corresponding to old age. Gender included two categories, male and female, and residence included two categories, urban and rural.

The data collected about the FPDs were location (maxilla and mandible); type according to the materials it was made of (metal–ceramic and metal–acrylic); FPD duration (representing the age of the FPD, considered until the current moment of the study), with the following categories: 0–5 years, 6–10 years, 11–15 years, 16–20 years, and over 20 years; the number of abutment teeth with the following categories: 1 abutment tooth, 2 abutment teeth, and up to 8 abutment teeth; number of intermediate elements starting with 0 intermediates in the case of joint crowns and up to 8 intermediate elements; the total number of elements of a restoration, with four categories: 2-element restorations, 3–4-element restorations, 5–7-element restorations corresponding to a hemiarch, and extensive restorations at the level of several sextants or on the entire arch with 8–16 elements; and the position of the pontic in relation to the abutment teeth with the following categories: conventional bridge (intercalated pontic), cantilever bridge (pontic in extension), and intercalated pontic associated with pontic in extension.

The data collected on FPD overload due to dental occlusion included the presence of an unbalanced occlusion plane based on clinical examination (yes/no), the presence of premature contacts and occlusal interferences (yes/no) based on clinical examination, as well as the presence of shortened dental arches (yes/no), also based on clinical examination.

The dental arch was considered shortened when a maximum of 20 teeth were present, the anterior region was intact, and a reduced number of posterior occlusal contacts were present [17].

Depending on the severity, complications of FPDs have been classified into 6 categories. This classification system has also been used in other clinical studies [18,19]:

- Grade 1 complications that can be corrected by occlusal adaptation or composite resin repairs without requiring replacement of the FPD [20]. Decementation of the FPD, unless it is due to a defect and the bridge can be cemented, is also considered a grade I complication.
- Grade 2 complications, where the FPD itself is still acceptable. However, the abutment tooth requires interventions or the use of a post and core.
- Grade 3 complications require replacement of the restoration only. The abutment tooth is acceptable at the abutment and root level. Marginal, technical, and aesthetic deficiencies of the FPD are included.
- In grade 4 complications, the FPD requires replacement, and the tooth structure of the abutment tooth is deficient. This includes caries of the abutment teeth and fractures, requiring augmentation of the abutment teeth structures.
- In grade 5 complications, the abutment teeth can no longer provide adequate support for the existing FPD due to extensive fractures, carious processes, periodontal damage, or other complications. Sometimes, extraction of the abutment teeth is required. Conventional FPD remains a reasonable option when other teeth are available for a redesigned restoration.
- Grade 6 complications are the most severe manifestation of FPD complications. The abutment teeth are compromised and replacement of the conventional FPD is no longer possible.

The data collected were entered into an Excel table and statistically processed.

### 2.5. Statistical Analysis

Initial data were organized in a table using Microsoft Excel 365 (San Francisco, CA, USA) and various categories and data series were determined. The subsequent statistical analysis was performed using Statistical Package for Social Sciences (SPSS) software application, version 26 (SPSS Inc., Armonk, NY, USA). Continuous variables were described as median values, following the evaluation of normality based on the Shapiro–Wilk test. Nominal and ordinal variables were described as frequencies and percentages and were analyzed using the chi-square test, for which the effect size was determined as the ratio between the square root of the chi-square computed value and the size of the study lot. Comparisons between the previously identified groups were performed using the Mann–Whitney U test (for whom the effect size, represented as *r*, was calculated as the ratio between the standardized test statistic z (represented as an absolute value) and the square root of the sample size (meaning the number of observations in the dataset)) and Kruskal–Wallis H test, followed by associated pairwise comparisons based on Dunn’s procedure, with the Bonferroni correction recommended for multiple comparisons. For this test, the effect size was denoted as η^2^ and was computed as the ratio between the test statistic H minus the number of groups plus 1 (H − k + 1) and the difference between the size of the study lot and the number of groups (n–k). The statistical significance threshold was considered to be 0.05.

## 3. Results

Following the application of the inclusion and exclusion criteria, 306 patients were included in the study and, for each of them, only one FPD was considered.

Regarding the general data of the patients studied, the maximum age was 84 years and the minimum was 36 years, with an average of 57.67 ± 8.66 years old. Most patients were included in the age group 46–60 years (156 patients, representing 50.98%), followed by the groups 61–75 years old (110 patients, 35.95%), 30–45 years old (34 patients, 11.11%), and 75+ years old (6 patients, 1.96%). Most patients lived in urban areas—230 patients (75.16%)—and 76 patients (24.84%) lived in rural areas.

The distribution of the patients by gender revealed that 208 patients were women, representing 67.97% of the entire study lot, and 98 were men, representing 32.03%. Females are predominant within the age groups 46–60 and 61–75, emphasizing statistically significant differences regarding the distribution of gender by age groups, *p* < 0.001, Table 1. The analysis by residence and FPD location revealed no statistically significant differences between females and males, *p* > 0.05 (Table 1).

The analysis of the bridges’ fabrication material revealed that 180 bridges (58.82%) were metal–ceramic and 126 bridges (41.18%) were metal–acrylic. Females have both types of bridges, distributed rather similarly, but there is a clear predominance of metal–ceramic prosthetics for males, 71.43%, compared to only 28.57% metal–acrylic ones. Overall, there is a statistically significant association between bridges’ material and gender, *p* = 0.002 (Table 2). Age group analysis also reflects a statistically significant association; young patients prefer metal–ceramic restorations, while elderly patients, with ages more than 60 years old, prefer metal–acrylic restorations, *p* < 0.001, Table 2. In terms of residence, almost three-quarters of the urban patients have metal–ceramic restorations, compared to 21.05% rural patients, indicating a statistical association, *p* < 0.001, Table 2.

The distribution of bridges by location indicated that 202 FPDs (66.01%) were located at the maxilla level, while 104 FPDs (33.99%) were located in the mandible. Metal–ceramic FPDs are predominant at the maxilla level, with almost two thirds of the total number of bridges, while the mandible FPD types are almost equal in percentages. The differences are important but not significant from a statistical point of view, *p* = 0.078 (Table 3). Metal–ceramic restorations have a lower duration (mostly up to 15 years) compared to metal–acrylic restorations, which last even more than 20 years, the differences between them being statistically significant (*p* < 0.001), Table 3.

Pontics of FPDs are mostly intercalated (83.33%), as well as more than half being metal–acrylic (65.08%); there are statistically significant differences at a cantilever level, where metal–acrylic pontics of FPDs are predominant (*p* = 0.001) (Table 3).

An unbalanced occlusion plane was observed in 248 patients (81.04%), the presence of premature contacts and occlusal interferences was recorded in 254 patients (83.00%), and shortened arches were recorded in half of the patients included in the study, 156 (50.98%). More than half of patients with shortened arcades (56.41%) have metal–acrylic restorations, while three quarters of patients without shortened arcades have metal–ceramic ones, leading to statistically significant differences between these groups of patients, *p* < 0.001 (Table 3).

The analysis of FPD duration reflected that most bridges with complications, 132 (43.14%), were between 6 and 10 years old, followed by bridges between 11 and 15 years old, 78 (25.49%).

The evaluation of the support of the dental bridges, namely the number of abutment teeth, highlighted that, for the 306 FPDs, there were 870 abutment teeth. Most of the FPDs had two abutment teeth, 168 (54.90%), followed by bridges with three abutment teeth, 68 (22.22%) (Table 4). The FPDs in the study lot comprised a total number of 616 intermediaries (Table 4). Most frequently, the bridges presented one intermediary, 116 bridges (37.90%), while 98 bridges presented two intermediaries (32.02%), Table 4.

Regarding the total number of elements of the dental bridges, they summed 1486 elements, with an average of 4.85 elements per FPD. Most FPDs had around 3–4 elements, 172 (56.20%), followed by bridges with 5–7 elements, 94 (30.71%), bridges with 8–16 elements, 32 (10.45%), and bridges with 2 elements, 8 (2.61%). The complete distribution is indicated in Table 4.

Depending on the position of the pontic relative to the abutment teeth, in 232 cases (75.81%) it was intercalated, in 38 bridges it was in extension (12.41%), and 36 bridges (11.76%) presented intercalated and extension pontics.

Data regarding the complications of FPDs showed a predominance of grade 6, with more than a third of FPDs being in this category. Around 15–18% were grouped in each of the following categories: grades 3, 4, and 5. The complete distribution is presented in Table 5.

A Kruskal–Wallis test was conducted to determine if there were differences in the number of elements that differed by their severity grade. Distributions of the number of elements were similar for all groups, as assessed by visual inspection of a boxplot. Median number of elements was statistically significantly different between the different grades of complication severity, χ^2^(3) = 33.986, *p* < 0.001. Subsequently, pairwise comparisons were performed using Dunn’s procedure. A Bonferroni correction for multiple comparisons was made with statistical significance accepted at the *p* < 0.0033 level. This post hoc analysis revealed statistically significant differences in the number of elements between grades 5 and 6, 5 and 4, and 2 and 6 (adjusted *p* < 0.05) but not between any other grade combinations.

The distribution of numerical variables reflecting the number of FPD elements, FPD duration, abutment teeth and intermediaries, as well as complications’ severity according to different parameters are described in Table 6 and Table 7.

The analysis of patients’ age reflected that elderly patients, 75+, have a median number of FPD elements equal to 5, while all other age groups have a median number of elements equal to 4; thus, no significant differences between age groups were identified with respect to the total number of bridge elements. Similar results were recorded for the number of abutment teeth and intermediaries, *p* > 0.05. Overall, as the age group increased, the median number of the numerical parameters from Table 6 also increased. A borderline *p* value was obtained for the severity grade, as patients above 60 years old mostly have high grades, *p* = 0.063. A statistically significant difference was observed for the FPD duration; patients above 75 years old have a median FPD duration of 15 years and patients above 61 years old have a median FPD duration of 13 years, clearly higher than younger patients with a median duration of 8-9 years old (*p* = 0.001), Table 6.

Gender analysis reflected that only FPD duration was statistically significant between females and males, as females have a median duration of 11.50 years, compared to males with a median of only 9 years old, *p* = 0.003. No other parameters yielded statistically significant results (Table 6).

Residence analysis also emphasized clear results; patients with urban residence had a smaller median number of bridge elements, a median lower severity grade, less intermediaries, and a lower FPD duration, compared to patients with rural residence, *p* < 0.001, Table 6. Only the median values of abutment teeth were similar for all patients, 2, no matter their residence, *p* = 0.737.

Metal–acrylic restorations have significantly higher FPD duration, with a median value of 15 years old, compared to only 9 years old for metal–ceramics restorations. Similarly, metal–acrylic restorations have a significantly higher number of intermediaries and more severe complication grades, *p* < 0.05. No differences were observed for the total number of elements (Table 7).

Location analysis showed significant differences for FPD duration, with median older duration corresponding to maxillary FPDs. Significant differences were also present for the total number of FPD elements and the number of abutment teeth, *p* < 0.05 (Table 7).

Older FPDs have a significantly higher number of elements, intermediaries, and severity grades, *p* < 0.05. No statistically significant differences were observed for the number of abutment teeth (Table 7).

The FPD position also seems to be important with respect to all numerical parameters analyzed in Table 7, with statistically significant differences between positions. Although all three positions have similar median numbers of elements, abutment teeth, and intermediaries, there are statistically significant differences between them, *p* < 0.001. Cantilever FPDs have the higher median duration (15 years old), compared to only 9 years for the intercalated pontics of FPDs. Cantilever pontics and mixed pontics (cantilever and intercalated) have the most severe complication grades, with the median value 6.

The presence of an unbalanced occlusion plane is significant only with respect to the severity grade, as patients with this feature have more severe complications, *p* = 0.002, Table 7. Patients with occlusal interferences had a significantly lower FPD median duration, 10 years, compared to 15 years for the other patients, *p* = 0.007, Table 7. Patients with shortened arches maintained their FPDs for a longer time period (median duration 13 years old), compared to a median duration of 9 years old for the other patients, despite the fact that they presented complications with a high severity grade.

## 4. Discussion

Tooth loss creates a set of disorders in the DMS and, to resolve these disorders, FPDs represent a reliable treatment option, used for decades to restore oral function and aesthetics [21]. FPDs have an important role in restoring occlusion, masticatory efficiency, and facial harmony [22,23].

The evolution of FPDs is a subject of significant clinical interest, as complications can vary depending on a multitude of factors. Among these, occlusal loads stand out as a determining factor in the overall success of a bridge. Occlusal loads refer to the forces exerted on dental bridges, during DMS functions such as mastication and swallowing, but also bruxism loads such as clenching and grinding [24,25]. Occlusal loads can have repercussions on FPDs and their biological support.

The present study was conducted in a group of 306 patients of both genders from urban and rural areas, with an average age of 57.67 years, who presented for treatment due to complications of FPDs. Statistical processing of the data related to the study group showed a greater interest of women compared to men in oral health, as well as a greater access to dental treatments for people from urban areas. This situation is explainable by the higher prevalence of oral diseases in women. A study conducted in Romania in 2017 showed a much higher prevalence of dental lesions in women, in a proportion of 2/3, a similar situation found in other studies [26], but there are also published studies emphasizing that dental lesions have a higher prevalence in men [27]. Regarding the influence of general factors on the evolution of FPDs, these could affect the survival of dental bridges and should be considered as follows: patient age and gender [28], smoking [29], and treatment provider [30]. The amplitude of occlusal forces is influenced by patient age and gender (male patients have higher occlusal forces than female patients, and young patients have higher occlusal forces than older patients) [31]. The location of the FPDs in the present study showed a greater concern for prosthetics in the maxilla, probably due to physiognomic considerations, but the complication rate is about the same for both arches. Studies related to the survival of maxillary versus mandibular FPDs showed no significant influence [21], while another study reported a significantly longer median survival time for mandibular FPD compared to maxillary ones [32].

Regarding the materials from which FPDs were made, the present study considered metal–ceramic and metal–acrylic bridges.

Nowadays, in Romania, in current prosthetic therapy, these two types of bridges are most frequently used. Metal–ceramic FPDs constitute the gold standard to which other types of bridges are compared in terms of aesthetics and resistance, and the equipment and production technology are well known to all dental technicians in Romania [33]. Metal–acrylic FPDs, on the other hand, are reimbursed by health insurance systems in Romania. For semi-physiognomic metal–acrylic elements, 60% is reimbursed by health insurance, the rest being the patients’ contribution.

The present study, by evaluating the duration of the bridges, shows a trend of abandoning metal–acrylic FPDs in favor of metal–ceramic ones.

Regarding the survival of metal–ceramic FPDs, a meta-analysis reported a survival rate of 94.7% after 5 years [34,35] and, for metal–acrylic FPDs with noble alloy frameworks, a failure rate of 4% after 5 years, 12% after 10 years, and 32% after 15 years was reported [36]. The present study showed that metal–ceramic FPDs had complications in a higher proportion than metal–acrylic FPDs with an average of complications that can be reported as failure of 7.84%. Therefore, the material from which FPDs are made can be considered as a potential factor that can affect their survival [33]. A study conducted in the UK showed a 72% survival rate of dental bridges without re-intervention at 10 years [30]. Significant factors influencing the low survival rate of FPDs included the type of bridge, patients with high treatment needs, and poorer oral status [30]. The selection of the appropriate material for FPDs has become more difficult due to the increasing variety of restorative materials. This selection of material is based on the available interocclusal space, aesthetic aspects (e.g., brightness value or translucency of neighboring teeth), as well as clinical evidence extracted from survival rates [37].

Regarding the extent of FPDs (number of abutment teeth and intermediaries), most FPDs in the study had two abutment teeth and one to two intermediaries, so FPDs with three to four elements. Associating the number of elements with the number of complications and their severity, it was found that, as the number of elements increases, the share of complications and their severity increase too, FPDs with four elements and those with more than four elements presenting complications of grades 5, 5, and 6.

Another study reported on the FPD duration and the number of abutment teeth and showed a significantly lower survival rate for extended bridges (5–11 units) compared to short-span bridges (3–4 units), after an average follow-up of 7.5 years [38]. A similar study compared two other types of FPDs: reduced FPDs (three to four elements) with extended FPDs (five to nine elements). After 20 years, the estimated survival rate was significantly higher for reduced-span bridges (70.8%) compared to extended bridges (52.8%) [39]. Bridges connect teeth with different functional values, and the stresses transmitted to the FPD are distributed differentially on the abutment teeth. In addition, a series of tilting and bending stresses occur at the level of FPD, which are accentuated in the case of occlusal imbalances and long-span bridges [40].

Furthermore, the presence of a large number of abutment teeth supporting an FPD could increase the complexity of the treatment and the risk of complications, but there is limited information in this regard [39]. In addition to the number of elements in the FPDs, another prognostic factor is the position of the pontic in relation to the abutment teeth. In the case of cantilever bridges, the stresses on the pontic exert a tipping effect on the bridge and abutment teeth [41]. In the present study, the severity of complications was much higher for cantilever FPDs. Other studies have also shown that the success and survival rates for cantilever FPDs are poorer than those for conventional FPDs and this is accompanied by frequent biological and technical complications [42].

Chai J., in 2005, compared conventional three-unit bridges with two-unit cantilever bridges 48 months after their fabrication and found no statistically significant differences [43]. It is considered that reducing the number and size of the extension and increasing the number of abutment teeth are essential for the evolution of cantilever FPDs. In addition, a balanced occlusion improves the results [44].

Dental occlusion plays an important role in the evolution of FPDs, influencing both functional stability and their durability over time [45,46]. Dental occlusion is represented by the contacts that occur between the teeth of the two arches during DMS functions [44]. Static occlusion refers to the relationship between the maxillary and mandibular teeth in the position of maximum intercuspation (MI), while dynamic or eccentric occlusion refers to this relationship between the teeth of the two arches during the exercise of DMS functions [47,48,49]. Occlusal forces are characterized by direction, amplitude, duration, and frequency. Excessive occlusal force is defined as an occlusal force that exceeds the reparative capacity of the marginal periodontium, leading to occlusal trauma and excessive loss of tooth structure [50]. Changes in the distribution of forces in the dental arches cause changes in the other components of the DMS to adapt to the new condition [10]. While balanced occlusal relationships provide stability, comfort, and functionality, dysfunctional occlusion can contribute to numerous complications [51]. Prosthetic treatment aims to restore or improve masticatory function, which depends on the distribution of occlusal contacts and the amplitude of forces [49,52,53]. An “ideal” occlusion that ensures optimal masticatory function has not yet been defined, but it is considered that there should be a maximum number of occlusal contacts in the position of MI, distributed bilaterally and ensuring the transmission of occlusal forces through an axial load of the posterior teeth [47,54]. Through prosthetic rehabilitation, it is recommended to restore the occlusal scheme that the patient previously presented [8]. If a single tooth makes premature contact during mandibular excursions, an unbalanced force distribution is created, which can lead to occlusal trauma [45]. The latter can include pain or discomfort, tooth mobility, progressive tooth wear, tooth or restoration fractures, and TMD [55]. Occlusal trauma resulting from these occlusal disharmonies can further aggravate periodontal conditions, compromising not only the restoration but also the health of the supporting structures. In addition to the distribution of occlusal contacts and the direction of occlusal loads according to the tooth implantation axis, other factors that influence the evolution of prosthetic restorations are represented by the amplitude of occlusal loads and their frequency. High muscle force can cause overloading of FPDs and increase the risk of technical and biological complications as occurs in bruxism [56]. Technical complications, such as loss of retention and material fracture, have been reported with greater frequency in patients with bruxism [57]. Patients with bruxism tend to have a higher prevalence of prosthetic complications, including a higher incidence of restoration fractures, tooth fracture, and a higher number of implant failures [58,59,60].

The role of excessive occlusal forces in bruxism remains controversial [56]. According to Nishigawa et al. [61], the amplitude of the maximum force detected in bruxism events is greater than the maximum voluntary force of occlusion during the day, having a destructive potential for teeth, periodontium, and dental restorations.

Patients with bruxism can exert considerable force on their teeth, and a large part of these may have a lateral component. Posterior teeth do not tolerate lateral forces as well as vertical forces on their long axes [6].

The present study evaluated the influence of occlusal factors on the severity of FPD complications, using a classification that is particularly indicative for the therapeutic approach to be adopted in each clinical situation [18,19]. According to the inclusion in this classification, grade 1 and grade 2 complications can be resolved without replacing the FPD, grade 3 and 4 complications require replacing the FPD, grade 5 complications require replacing the FPD and an additional number of abutment teeth, and, for grade 6 complications, FPD can no longer be performed, requiring removable or implant-supported prostheses.

According to the results of this study, most of the patients presented complications of grades 4, 6, 3, and 5. The explanation could be given by the financial situation, difficult access to treatment, and poor medical education.

Considering the association between these complications and occlusal factors, we believe that proper attention is not given to occlusal preprosthetic treatments. The explanations could be the same—financial, namely, the fact that most of the patients cannot financially support a complete rehabilitation of the dental arches and must keep the existing restorations. Correct prosthetic rehabilitation should ensure FPDs of functional occlusal relationships in harmony with both dynamic and static conditions for a good prognosis and the support of prosthetic restorations. This study draws attention to the need for preprosthetic occlusal balancing, even after definitive cementation of prosthetic restorations. In some situations, it is necessary to remove more recent prosthetic restorations from the oral cavity that disrupt the occlusal scheme, with great financial efforts. For all situations it is recommended to maintain the initial occlusal scheme and avoid occlusion changes. In the case of patients with high occlusal overloads, it is recommended to make an occlusal mouthguard that will protect the prosthetic restorations and will help to monitor occlusal loads, as well as periodic controls [62].

The limitations of this study were represented by the number of patients, the lack of knowledge of the initial functional value of the abutment teeth, and the fact that the FPDs were performed by several dental laboratories with different particularities of the metal framework and veneering materials.

## 5. Conclusions

This study evaluated the complications of metal–ceramic and metal–acrylic FPDs in terms of their severity associated with occlusal disharmonies. The highest number and the most severe complications were recorded for metal–acrylic FPDs, over 16 years old, in elderly patients from rural areas. The increased number of elements of FPDs was associated with greater severity of complications.

All considered occlusal factors were associated with a high severity of FPD complications, but the most harmful factors that caused excessive overloads were shortened arches and cantilever FPDs. This study draws attention to the role of balanced occlusal relationships in the evolution of FPDs and full arch prosthetic rehabilitation.

## Figures and Tables

**Table 1:** Distribution of the study lot by gender and demographic characteristics.

Parameter	Category	Females	Males	Total	*p* *
		208 (67.97%)	98 (32.03%)	306 Patients	
Age group (years old)	30–45	16 (47.06%)	18 (52.94%)	34 (100%)	<0.001 *^,#^ *r* = 0.248
		7.69%	18.37%		
	46–60	118 (75.64%)	38 (24.36%)	156 (100%)	
		56.73%	38.78%		
	61–75	74 (67.27%)	36 (32.73%)	110 (100%)	
		35.58%	36.73%		
	75+	0 (0%)	6 (100%)	6 (100%)	
		0%	6.12%		
Residence	Urban	158 (68.7%)	72 (31.3%)	230 (100%)	0.638 ** ω = 0.027
		75.96%	73.47%		
	Rural	50 (65.79%)	26 (34.21%)	76 (100%)	
		24.04%	26.53%		
Location	Maxillary	134 (66.34%)	68 (33.66%)	202 (100%)	0.392 ** ω = 0.049
		64.42%	69.39%		
	Mandible	74 (71.15%)	30 (28.85%)	104 (100%)	
		35.58%	30.61%		

* Mann–Whitney U test. ** Chi square test. ^#^ Statistically significant. The values in grey represent the sum by columns.

**Table 2:** Distribution of the study lot by restoration type and demographic characteristics.

Parameter	Category	Metal–Ceramic	Metal–Acrylic	Total	*p*
		180 (58.82%)	126 (41.18%)	306 FPDs	
Gender	Females	110 (52.88%)	98 (47.12%)	208 (100%)	0.002 *^,#^ ω = 0.176
		61.11%	77.78%	67.97%	
	Males	70 (71.43%)	28 (28.57%)	98 (100%)	
		38.89%	22.22%	32.03%	
Age group (years old)	30–45	32 (94.12%)	2 (5.88%)	34 (100%)	<0.001 **^,#^ *r* = 0.706
		17.78%	1.59%	11.11%	
	46–60	100 (64.1%)	56 (35.9%)	156 (100%)	
		55.56%	44.44%	50.98%	
	61–75	48 (43.64%)	62 (56.36%)	110 (100%)	
		26.67%	49.21%	35.95%	
	75+	0 (0%)	6 (100%)	6 (100%)	
		0%	4.76%	1.96%	
Residence	Urban	164 (71.3%)	66 (28.7%)	230 (100%)	<0.001 *^,#^ ω = 0.441
		91.11%	52.38%	75.16%	
	Rural	16 (21.05%)	60 (78.95%)	76 (100%)	
		8.89%	47.62%	24.84%	

* Chi square test. ** Mann–Whitney U test. ^#^ Statistically significant. The values in grey represent the sum by columns.

**Table 3:** Distribution of the study lot by FPD type and characteristics.

Parameter	Category	Metal–Ceramic	Metal–Acrylic	Total	*p*
		180 (58.82%)	126 (41.18%)	306 FPDs	
FPD location	Maxillary	126 (62.38%)	76 (37.62%)	202 (100%)	0.078 * ω = 0.101
		70%	60.32%	66.01%	
	Mandible	54 (51.92%)	50 (48.08%)	104 (100%)	
		30%	39.68%	33.99%	
FPD duration (years old)	0–5	22 (91.67%)	2 (8.33%)	24 (100%)	<0.001 **^,#^ *r* = 0.665
		12.22%	1.59%	7.84%	
	6–10	120 (90.91%)	12 (9.09%)	132 (100%)	
		66.67%	9.52%	43.14%	
	11–15	28 (35.9%)	50 (64.1%)	78 (100%)	
		15.56%	39.68%	25.49%	
	16–20	10 (17.86%)	46 (82.14%)	56 (100%)	
		5.56%	36.51%	18.3%	
	20+	0 (0%)	16 (100%)	16 (100%)	
		0%	12.7%	5.23%	
Pontic position	Cantilever	14 (36.84%)	24 (63.16%)	38 (100%)	0.001 *^,#^ ω = 0.213
		7.78%	19.05%	12.42%	
	Intercalated	150 (64.66%)	82 (35.34%)	232 (100%)	
		83.33%	65.08%	75.82%	
	Cantilever and	16 (44.44%)	20 (55.56%)	36 (100%)	
	intercalated	8.89%	15.87%	11.76%	
Unbalanced occlusion plane	Yes	140 (56.45%)	108 (43.55%)	248 (100%)	0.081 * ω = 0.100
		77.78%	85.71%	81.05%	
	No	40 (68.97%)	18 (31.03%)	58 (100%)	
		22.22%	14.29%	18.95%	
Occlusal interferences	Yes	150 (59.06%)	104 (40.94%)	254 (100%)	0.856 * ω = 0.010
		83.33%	82.54%	83.01%	
	No	30 (57.69%)	22 (42.31%)	52 (100%)	
		16.67%	17.46%	16.99%	
Shortened arcades	Yes	68 (43.59%)	88 (56.41%)	156 (100%)	<0.001 *^,#^ ω = 0.316
		37.78%	69.84%	50.98%	
	No	112 (74.67%)	38 (25.33%)	150 (100%)	
		62.22%	30.16%	49.02%	

* Chi square test. ** Mann–Whitney U test. ^#^ Statistically significant. The values in grey represent the sum by columns.

**Table 4:** Distribution of FPDs according to abutment teeth and intermediaries.

Abutment Teeth	0	1	2	3	4	5	6	7	8
Number of FPDs	-	8 (2.50%)	168 (54.90%)	68 (22.22%)	28 (9.15%)	8 (2.50%)	18 (5.88%)	2 (0.65%)	6 (1.96%)
**Intermediaries**	**0**	**1**	**2**	**3**	**4**	**5**	**6**	**7**	**8**
Number of FPDs	14 (4.57%)	116 (37.90%)	98 (32.02%)	46 (15.03%)	16 (5.22%)	2 (0.65%)	10 (3.26%)	0 (0%)	4 (1.20%)
**Total**	14	124	266	114	44	10	28	2	10

**Table 5:** Distribution of FPDs according to the severity of complications.

Grade	Grade 1	Grade 2	Grade 3	Grade 4	Grade 5	Grade 6	*p* *
FPDs	12 (3.92%)	24 (7.84%)	48 (15.68%)	56 (18.30%)	48 (15.68%)	118 (38.56%)	<0.001 ^#^ *η*^2^ = 0.097
Median number of elements	3.50	3.50	4.00	4.50	3.50	4.00	

* Kruskal–Wallis H test. ^#^ Statistically significant.

**Table 6:** Distribution of FPDs and associated characteristics according to the demographic parameters.

Parameter	Category	FPD Elements	FPD Duration (Years Old)	Abutment Teeth	Intermediaries	Grade
		Median Values				
Age group (years old)	30–45	4.00	8.00	2.00	1.50	4.00
	46–60	4.00	9.00	2.00	2.00	4.50
	61–75	4.00	13.00	2.00	2.00	5.00
	75+	5.00	15.00	3.00	2.00	5.00
	*p* *	0.467 *η*^2^ = 0.002	<0.001 ^#^ *η*^2^ = 0.105	0.699 *η*^2^ = 0.005	0.465 *η*^2^ = 0.001	0.063 *η*^2^ = 0.014
Gender	Females	4.00	11.50	2.00	2.00	4.50
	Males	4.00	9.00	2.00	2.00	5.00
	*p* **	0.191 *r* = 0.075	0.003 ^#^ *r* = 0.172	0.595 *r* = 0.030	0.152 *r* = 0.306	0.365 *r* = 0.288
Residence	Urban	4.00	9.00	2.00	1.00	4.00
	Rural	5.00	15.50	2.00	2.00	6.00
	*p* **	<0.001 ^#^ *r* = 0.211	<0.001 ^#^ *r* = 0.346	0.737 *r* = 0.019	<0.001 ^#^ *r* = 0.306	<0.001 ^#^ *r* = 0.288

* Kruskal–Wallis H test. ** Mann–Whitney U test. ^#^ Statistically significant. The values in grey represent the sum by columns.

**Table 7:** Associations between FPDs characteristics.

Parameter	Category	FPD Elements	FPD Duration (Years Old)	Abutment Teeth	Intermediaries	Grade
				Median Values		
FPD type	Metal–ceramics	4.00	9.00	2.00	1.00	4.00
	Metal–acrylic	4.00	15.00	2.00	2.00	5.50
	*p* *	0.329 *r* = 0.056	<0.001 ^#^ *r* = 0.675	0.035 ^#^ *r* = 0.120	<0.001 ^#^ *r* = 0.236	0.001 ^#^ *r* = 0.184
FPD location	Maxillary	4.00	12.00	2.00	2.00	5.00
	Mandible	4.00	9.00	2.00	2.00	4.50
	*p* *	0.005 ^#^ *r* = 0.160	0.002 ^#^ *r* = 0.176	< 0.001 ^#^ *r* = 0.218	0.593 *r* = 0.031	0.154 *r* = 0.082
FPD duration	0–5	4.00	-	2.00	1.50	2.50
(years old)	6–10	4.00	-	2.00	2.00	4.00
	11–15	4.00	-	2.00	1.00	5.00
	16–20	5.00	-	2.00	2.00	6.00
	20+	5.00	-	2.00	3.00	6.00
	*p* **	0.008 ^#^ *η*^2^ = 0.036	-	0.574 *η*^2^ = 0.001	<0.001 ^#^ *η*^2^ = 0.077	<0.001 ^#^ *η*^2^ = 0.175
Pontic position	Cantilever	4.00	15.00	2.00	1.00	6.00
	Intercalated	4.00	9.00	2.00	2.00	4.00
	Cantilever and intercalated	4.00	14.00	2.00	2.00	6.00
	*p* **	<0.001 ^#^ *η*^2^ = 0.048	<0.001 ^#^ *η*^2^ = 0.069	0.021 ^#^ *η*^2^ = 0.019	<0.001 ^#^ *η*^2^ = 0.051	<0.001 ^#^ *η*^2^ = 0.079
Unbalanced occlusion plane	Yes	4.00	10.00	2.00	2.00	5.00
	No	4.00	12.00	2.00	2.00	4.00
	*p* *	0.418 *r* = 0.046	0.926 *r* = 0.005	0.994 *r* = 0.001	0.163 *r* = 0.080	0.002 ^#^ *r* = 0.180
Occlusal interferences	Yes	4.00	10.00	2.00	2.00	5.00
	No	5.00	15.00	2.00	2.00	4.00
	*p* *	0.413 *r* = 0.047	0.007 ^#^ *r* = 0.154	0.949 *r* = 0.004	0.931 *r* = 0.005	0.330 *r* = 0.056
Shortened arches	Yes	4.00	13.00	2.00	2.00	6.00
	No	4.00	9.00	2.00	2.00	4.00
	*p* *	0.078 *r* = 0.101	< 0.001 ^#^ *r* = 0.321	0.911 *r* = 0.006	0.005 ^#^ *r* = 0.162	< 0.001 ^#^ *r* = 0.455

* Mann–Whitney U test. ** Kruskal–Wallis H test. ^#^ Statistically significant. The values in grey represent the sum by columns.

## Data Availability

The authors declare that the data of this research are available from the correspondence authors upon reasonable request.

## Abbreviations

The following abbreviations are used in this manuscript:

DMS	Dento-Maxillary System
FPD	Fixed Prosthetic Denture
